# National

**Published:** 1897-02

**Authors:** 


*National.*—The agreement just effected between the Secretary of Agriculture and the Canadian Minister of Agriculture relative to the quarantine of animals passing from one country to another, provides in substance that each country shall accept the veterinary certificate of the other as final, and that they shall keep each other informed of any outbreaks of contagious diseases; that a quarantine of from ten to fifteen days shall be imposed on cattle, ruminants or swine, coming from European countries in which pleuro-pneumonia, or foot-and-mouth disease exists; that breeding cattle shall have certificates of freedom from tuberculosis, or submit to a week's quarantine, and that cattle coming from feeding or stock-ranches must likewise have certificates of freedom from disease of all kinds (except tuberculosis) and exemption of their districts from disease. Provision is made for the admission in bond of cattle in transit at the ports of both countries, and for the regulation of the transit of animals on railways. Sheep may be admitted subject to inspection, with certificates of exemption from scab of the district whence they come, subject to slaughter if disease appears. They are also accorded privileges of passage in bond through ports without inspection. Swine for slaughter may pass without inspection to bonded slaughter-houses, or when properly certified as part of a settler's effects. Liberal rules are laid down for the entry of horses into either country, and particularly for those belonging to Indian tribes and travelers.

